# Lower Extremity Nodules After Spelunking in Mexico

**DOI:** 10.7759/cureus.39908

**Published:** 2023-06-03

**Authors:** Pranvera Sulejmani, Luke Wallis, Anas Alabkaa, Aadil Ahmed

**Affiliations:** 1 Dermatology, Rush University Medical Center, Chicago, USA; 2 Dermatology, University of Mississippi Medical Center, Jackson, USA; 3 Pathology, Rush University Medical Center, Chicago, USA; 4 Dermatopathology, Rush University Medical Center, Chicago, USA

**Keywords:** non-tuberculous mycobacterium, acid-fast bacillus, derm path, skin nodule, aquatic infections

## Abstract

*Mycobacterium marinum *is a non-tuberculous mycobacterium that presents as a nodular granulomatous disease. The bacillus can infect humans when broken skin is exposed to a contaminated aquatic environment. *M. marinum *infections are usually isolated to the skin and soft tissues and can spread in a lymphatic distribution. A 26-year-old male cut his right ankle while spelunking in Tulum, Mexico. He presented to his primary care physician three months after he sustained the laceration with a nonhealing wound on the right lateral posterior ankle. Examination of the lesion demonstrated erythematous, violaceous, and hyperpigmented indurated plaques with satellite lesions noted at the right medial, posterior, and lateral ankle. The lesion characteristics raised initial suspicion for an invasive fungal infection. Biopsy of the lesion demonstrated epidermal ulceration covered by neutrophilic serum, marked underlying dermal acute inflammation, and granulation tissue. A mild perivascular, predominantly lymphocytic infiltrate was present in the deep dermis with no evidence of granuloma. Acid-fast bacilli culture plated onto chocolate agar confirmed the species *M. marinum.*

## Introduction

*Mycobacterium marinum* is a non-tuberculous mycobacterium affecting 0.27 cases per 100,000 inhabitants that presents as a nodular granulomatous disease [1]. The bacillus typically causes a tuberculosis-like illness in aquatic organisms but can infect humans when damaged skin is exposed to a contaminated aquatic environment. *M. marinum* infections are usually isolated to the skin and soft tissue in immunocompetent individuals and spread in a sporotrichoid distribution [1].

This article was previously presented as a poster abstract at the 2022 American Society of Dermatopathology Annual Scientific Meeting on October 22, 2022.

## Case presentation

**Figure 1:**
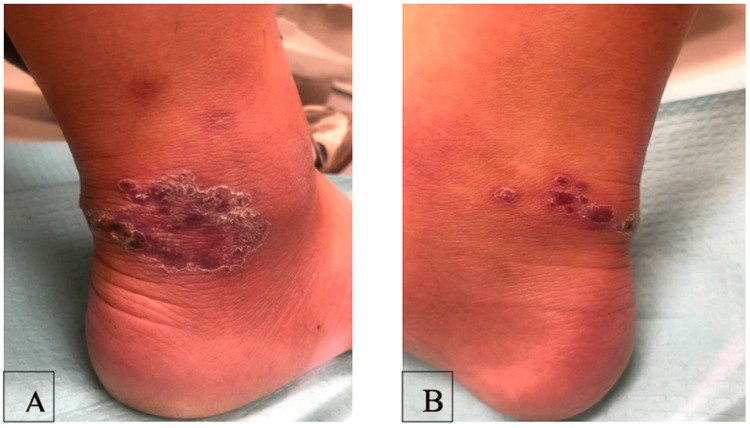
Clinical images of the right ankle. (A) Nodular lesion on the right lateral ankle caused by *Mycobacterium marinum*. (B) Nodular lesion on the right posteromedial ankle caused by *Mycobacterium marinum.*

A 26-year-old male with no significant past medical history sustained a small laceration on his right ankle while spelunking in Tulum, Mexico. Three months after the initial injury, he presented to his primary care physician with a nonhealing wound on his right ankle. Examination of the lesion demonstrated erythematous-to-violaceous indurated papules coalescing into plaques (Figure 1). The lesion raised suspicion for infection, and a biopsy and tissue culture for aerobic and anaerobic bacteria, atypical mycobacteria, and fungus was performed.

**Figure 2:**
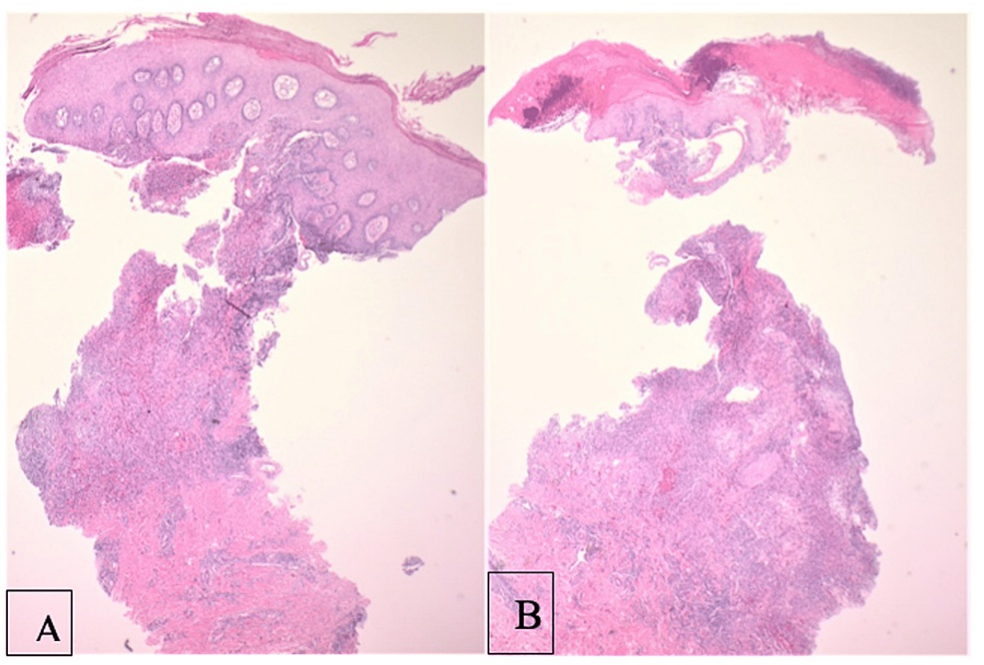
Hematoxylin and eosin stain low-power images. Demonstrates ulceration and epidermal hyperplasia. Original magnification 20×.

**Figure 3:**
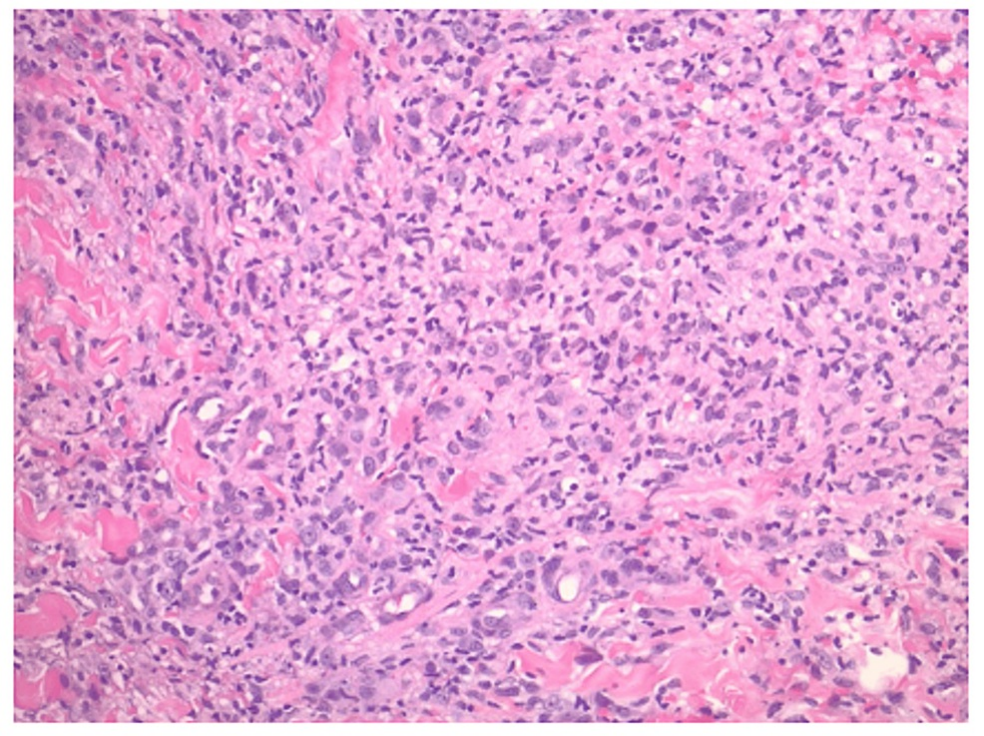
Hematoxylin and eosin stain of deeper dermis infected by Mycobacterium marinum. Demonstrates lymphohistiocytic infiltrate. Original magnification 200×.

Biopsy demonstrated skin with ulceration, neutrophilic serum, and adjacent reactive epidermal hyperplasia with an underlying neutrophil-rich superficial dermal infiltrate (Figure 2). In the deep dermis, the infiltrate was lymphohistiocytic without evidence of granuloma formation (Figure 3). Periodic acid-Schiff, Grocott’s methenamine silver, and Fite stains were negative for microorganisms. Fluorochrome staining was also negative for acid-fast bacilli; however, acid-fast bacilli culture plated onto chocolate agar confirmed the presence of *M. marinum*.

## Discussion

*M. marinum* is an endemic fish pathogen that is found in an assortment of aquatic environments such as natural water bodies, swimming pools, fish tanks, and aquariums [2]. *M. marinum* infections are indolent with an intermediate incubation period of 16 days [3]. The infection normally appears as a solitary papulonodular lesion on an extremity that has undergone preceding trauma or abrasion in an aquatic setting [4]. Over time, the lesion progresses and enlarges into violaceous nodular plaques that may ulcerate [4]. The lesions are typically painless and are not commonly associated with systemic symptoms [4]. However, the infection can spread to deeper structures and cause tenosynovitis, arthritis, and osteomyelitis [5]. Though the lesions can be self-limiting and heal spontaneously, *M. marinum* disease can take on a sporotrichoid pattern in about 20% of cases in which the local inoculation site is associated with nodular or ulcerating lesions that spread proximally up the lymphatics and to regional lymph nodes [4].

*M. marinum* infections may be confused with other differentials such as leishmaniasis, histoplasmosis, coccidioidomycosis, blastomycosis, and other fungal infections. Evaluation should include patient history, risk factors, duration of disease, site and morphology of lesions, as well as medical history [3]. Diagnosis of* M. marinum* can be challenging due to the difficulty in culturing the bacteria and its nonspecific clinical polymorphism [6]. Cultures usually take several weeks, oftentimes delaying the diagnosis.

Diagnosis is confirmed with bacterial growth in acid-fast bacilli culture at 25-30°C and biopsy. The histology shows suppurative inflammation in acute cases and mixed granulomatous inflammation with scattered multinucleated giant cells and neutrophils in long-standing cases [5]. Other common findings include epidermal changes, such as acanthosis and pseudoepitheliomatous hyperplasia [3] with or without ulceration. Acid-fast stains can highlight the organisms in most cases but can also be negative. Culture and bacterial polymerase chain reaction studies in such suspicious cases can be useful for confirmation.

Treatment of *M. marinum *infection is typically prolonged, requiring months of antibiotics to attain clearance. *M. marinum* can be particularly difficult to treat as an organism due to increasing multidrug resistance [6]. In cases limited to superficial cutaneous infection, monotherapy is usually effective [7]. Clarithromycin, doxycycline, minocycline, or trimethoprim-sulfamethoxazole monotherapy for three months has shown efficacy [3]. For severe infections, a combination of rifampin and ethambutol can be used [3].

This patient was empirically started on 100 mg of doxycycline twice daily. Two months into treatment, the erythema and tenderness of the lesions improved and began to heal without ulceration, necrosis, or discharge. Four months into treatment, the lesions continued to improve and the appearance of new lesions decreased significantly. The patient was counseled to stop doxycycline therapy after five months of treatment or follow-up if new lesions occurred. His treatment course lasted for five months.

## Conclusions

The primary form of inoculation by* M. marinum* is injury followed by exposure to contaminated marine environments. *M. marinum* primarily affects the superficial skin of extremities and classically appears as a nodular granulomatous disease. Though the infection may be self-limited in some individuals, the lesion has been reported to progress to a sporotrichoid pattern in others. Clearance of the infection can be achieved with monotherapy but may require polytherapy if it is severe and extends beyond a superficial cutaneous infection. This case demonstrates a classic history, clinical presentation, and histopathology of a rare infection in the United States.
